# Quantum dot-like excitonic behavior in individual single walled-carbon nanotubes

**DOI:** 10.1038/srep37167

**Published:** 2016-11-16

**Authors:** Xu Wang, Jack A. Alexander-Webber, Wei Jia, Benjamin P. L. Reid, Samuel D. Stranks, Mark J. Holmes, Christopher C. S. Chan, Chaoyong Deng, Robin J. Nicholas, Robert A. Taylor

**Affiliations:** 1 College of Big Data and Information Engineering, Guizhou University, Huaxi, Guiyang, 550025, P. R. China; 2 Department of Physics, University of Oxford, Clarendon Laboratory, Parks Road, Oxford, OX1 3PU, UK

## Abstract

Semiconducting single-walled carbon nanotubes are one-dimensional materials with great prospects for applications such as optoelectronic and quantum information devices. Yet, their optical performance is hindered by low fluorescent yield. Highly mobile excitons interacting with quenching sites are attributed to be one of the main non-radiative decay mechanisms that shortens the exciton lifetime. In this paper we report on time-integrated photoluminescence measurements on individual polymer wrapped semiconducting carbon nanotubes. An ultra narrow linewidth we observed demonstrates intrinsic exciton dynamics. Furthermore, we identify a state filling effect in individual carbon nanotubes at cryogenic temperatures as previously observed in quantum dots. We propose that each of the CNTs is segmented into a chain of zero-dimensional states confined by a varying local potential along the CNT, determined by local environmental factors such as the amount of polymer wrapping. Spectral diffusion is also observed, which is consistent with the tunneling of excitons between these confined states.

Carbon nanotubes (CNTs) act like single layers of carbon atoms rolled up from graphene sheets. The strong confinement to one dimension (1D) leads to unique electronic and optical properties^1^. Intense study has been focused on the photoluminescence (PL) from semiconducting single-walled CNTs since a method to prevent bundling of CNTs was discovered in 2002^2^. The existence of highly mobile excitons in CNTs is of great interest for CNT-based applications such as optical sensing^3^,^4^,^5^. Experimental observation of single CNT PL is challenging due to the low fluorescence yield and a strong structural (chirality) dependence of their optical properties^6^. Recently, the signatures of quantum light from individual CNTs were demonstrated^7^,^8^,^9^, attributed to the localization of excitons which form quantum dot-like (QD-like) states^10^. The existence of QD-like states has been observed in single CNT electronic transport measurements^11^ and near field optical spectroscopy^12^. To expand on this idea, a systematic study on QD-like excitonic behavior in CNTs is necessary.

A CNT’s chirality defines its atomic and electronic structure and thus the chirality-dependent CNT separation is a key process during sample preparation^13^. Several methods have been adopted to successfully select and separate individual semiconducting CNTs, including micelle encapsulation^6^, single as-grown suspended nanotubes^14^ and DNA surfactant coated nanotubes^15^. In the present study, we use a conjugated polymer to select nanotubes through non-covalent force of the polymer backbone on the CNT’s surface^16^. Polymeric molecules in general are composed of large molecules covalently bonding to their nearest neighbors. In the case of CNTs, repeated structures of *sp*^2^ hybridized carbon atoms in the form of *π*-conjugated polymers yield stable compounds, and thus give rise to the high selectivity of cross linked aromatic polymer towards certain CNT species in solution^17^. Figure 1 shows the chemical structure of poly[9,9-dioctylfluorene] (PFO) and an illustration of a PFO-nanotube compound. When dissolved in an O-xylene solution, the polymer wraps nanotubes selectively and thereby debundles them, producing an environment closer to an intrinsic nanotube for subsequent studies.

## Results

### Ultranarrow linewidth

**Figure 1:**
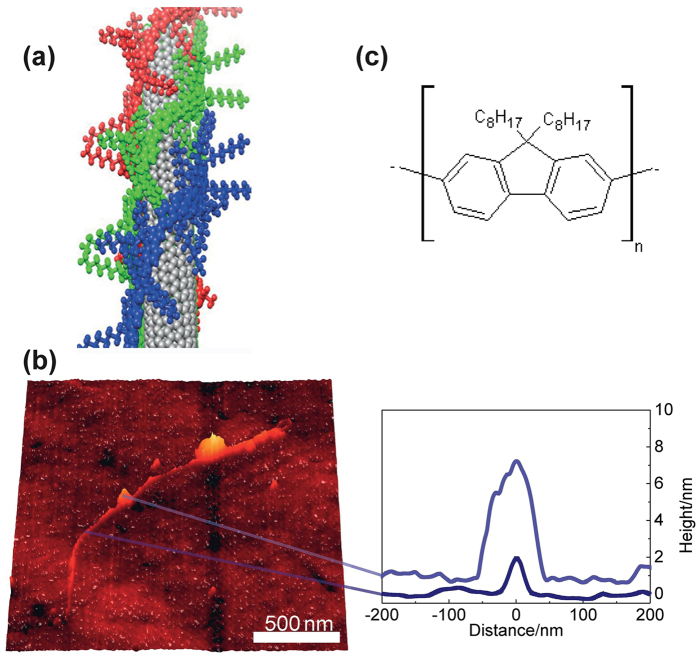
(**a**) Drawing of an (8, 6) CNT wrapped by a monolayer of PFO. The backbone polymer atoms are colored. The chemical structure of PFO is shown in (**c**). (**b**) An AFM image of a single CNT. The bright spots indicate the existence of aggregate polymer crystals. The inset shows the height profile of nanocrystals and nanotubes, with an 8 nm discontinuity measured.

The polymer surfactant CNT samples were prepared using methods described elsewhere^17^. Some polymers were found to be able to wrap fully around a CNT as shown in Fig. 1(a) and the rebundling rate was found to be low. The HiPCO CNTs were then dispersed on quartz wafers, to provide individualized spatially isolated CNTs. Figure 1(b) shows a typical AFM image of a dispersed CNT. An average CNT diameter of ~1.5 nm is measured, and is in good agreement with HiPCO single-walled carbon nanotubes wrapped in polymer^18^. Note the bright yellow spots visible in the AFM image in Fig. 1(b) are polymer aggregate crystals with a typical height of 5–15 nm^17^,^19^. In one dimensional materials, long lived excitons are highly mobile and these polymer crystals might be able to trap a charge in the close vicinity of the emitting states.

**Figure 2:**
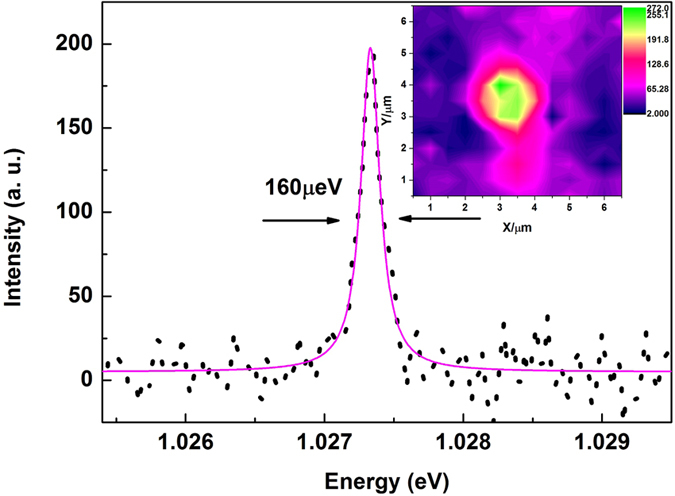
PL from an individual CNT with a FWHM of 160 *μ*eV. The dots are experimental data whilst the violet line is a Lorentzian fit. The inset is a 6 *μ*m by 6 *μ*m spatial map.

A striking observation from our experiments is the exceptionally narrow linewidth seen in the emission from the CNTs at cryogenic temperatures. In our setup, we observed emission spectra with width ranging from less than 200 *μ*eV to ~1 meV. The linewidth is independent of excitation power density when the excitation power is low (below 0.15 mW/*μ*m^2^), which implies that we have reached the intrinsic regime of individual carbon nanotubes^20^. This 160 *μ*eV full-width at half-maximum (FWHM) emission, as shown in Fig. 2, is amongst the narrowest linewidths of surfactant CNTs ever reported^21^,^22^. The inset of Fig. 2 shows a spatial photoluminescence map of a single CNT. We applied a sub-micron precision piezoelectric stage which helps us locate the individual CNTs accurately. We can use the linewidth measured to estimate an E_11_ exciton lifetime-broadening of over 10 ps, which is longer than some previously reported lifetimes^6^,^23^,^24^. This improved dephasing time in our PFO surfactant CNTs can be ascribed to the protection given by the PFO to the excitons from the local dielectric environment along the tube axis. As found in magnetic field studies^20^ that the weakly confined free excitons emit from localized states and as calculated in ref. 5 using suspended nanotubes, localization is responsible for enhancing dephasing correlated narrow line width by inhibiting the role of inhomogeneity induced diffusive line broadening. This decoupling from the environment could open the way for new studies on low dimensional quantum materials.

### State filling effect

**Figure 3:**
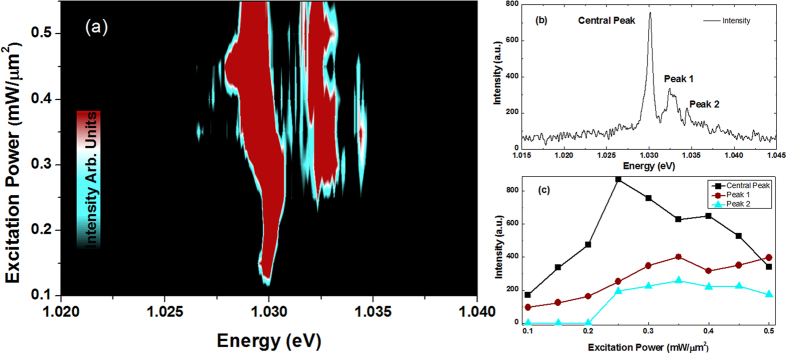
Power dependence measurements for an individual (8, 6) CNT. (**a**) Plot of excitation power against photon energy. New states emerge with increasing excitation energy. Colors changing from black to green to red indicate an increase in peak intensity. (**b**) A spectrum showing the state filling effect in an (8, 6) CNT. (**c**) Plot of emission intensity against excitation power for each peak from (**a**) showing saturation effect.

Further support for the picture of emission from weakly localized states is given by the observation of a state filling (SF) effect in the CNT emission. SF phenomenon is well known in systems with confined discrete energy states such as in InAs quantum dots (QDs)^25^. When lower energy states are populated with a certain number of excitons, the subsequent excitons must then occupy higher energy states due to the Pauli principle^26^. In CNTs, the quasi-1D electronic structure differs from QDs in that excitons are mobile along the tube direction and thereby a new multiexciton ground state could be created at higher energy^27^. However, if somehow the exciton path is blocked, then the exciton is localized and lower dimensional effects such as state filling become important. In Fig. 3, a series of spectra taken from individual (8, 6) CNTs are shown as a function of excitation power. In the low power regime (0.25 mW/*μ*m^2^ and below), only one sharp peak with ~1 meV FWHM is observed. When the excitation power is increased, a new peak about 2 meV higher in energy than the main peak starts to emerge (peak 1 in Fig. 3(b)). A further increase of excitation power results in peak 2 at still higher energy. The saturation effect showing in Fig. 3(c) and approximate equidistance of the emission features are similar to that seen in many self-assembled QDs^28^. This behaviour is typical for many PFO-wrapped nanotubes.

## Discussion

### The Ohmic model

Photoluminescence quenching sites and inhomogeneities have been suggested as two main broadening mechanisms in CNTs. In our case, we have a highly symmetric and ultra narrow line shape as shown in Fig. 2. Furthermore, the intrinsic regime not only demands a prolonged spontaneous emission lifetime (T1 time), but also requires a rather long exciton dephasing time. We demonstrate here that the polymer wrapping is fundamentally important as it protects the CNTs from being affected by the environment while the polymer crystals can act as localization centres^22^. The other two extrinsic candidates that affect the short T2 time are pure dephasing coupling to acoustic phonons and spectral diffusion. We discuss here all three proposed broadening mechanisms and demonstrate that they all affect the T2 time within their own scenarios. Firstly, it is consistent with the scenario in ref. 5, attributed to intrinsic exciton dephasing. Furthermore, the aggregated polymer crystals shown in Fig. 1(b) are considered to be able to separate and localize the mobile excitons. These localized excitons are able to form a chain of one-dimensional QD-like states linked by carriers transported along the CNT. The confinement is therefore governed by the interaction between excitons with 1D acoustic phonons, and can thus be treated theoretically using an independent boson model^29^. We assume a harmonic confinement localizes the exciton in CNTs with well-defined energy splitting 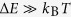 and that this system is independent from the surrounding environment. We have the exciton – phonon coupling matrix elements g_*j*_(q) as the product of the exciton form factor *F* ^exc^ and the bulk coupling matrix element *G*. We can then fit the experimental lineshapes as the mirror image of the absorption spectrum after a Fourier transformation^29^. Using an exciton confinement length *σ* = 3 nm and tube parameters *L* = 1500 nm with 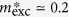, we obtain an excellent fit to the PL from the PFO-HiPCO CNTs as shown in supplementary information (SI). This model therefore works well for strongly confined nanotubes, and is equivalent to the Ohmic model used by Sarpkaya *et al.*^30^. However, the SF effect data cannot be fitted quantitatively because of the presence of higher energy peaks. This is when a non-Ohmic model is required, and implies that the extrinsic localized phonons play an important role as indicated by the broader asymmetric exciton spectra shown in the central peak of Fig. 3(b). Sarpkaya *et al.* used a non-Ohmic model to fit the zero phonon line (ZPL) with a side peak^30^. Nonetheless both the ohmic and non-ohmic models provide evidence to support the assertion that the luminescence originates from QD-like confinement, as they successively match the exciton spectra and ZPL width, under different circumstances. Note the red shift with increasing excitation power in Fig. 3 is a sign of quantum confined Stark effect (QCSE) as in 0D quantum emitters^31^,^32^. Similarly we observed spectra with two peaks emerging above the original peak and an extra one at lower energy (shown in the SI), again indicating exciton localization. This is consistent with the activation of the exchange split dark exciton state at non-zero field which occurs due to the coupling of the two degenerate valleys at the *K* and *K*′ points and has been shown to give a splitting of order 2 meV for the free excitons^20^.

### Spectral diffusion and localization

**Figure 4:**
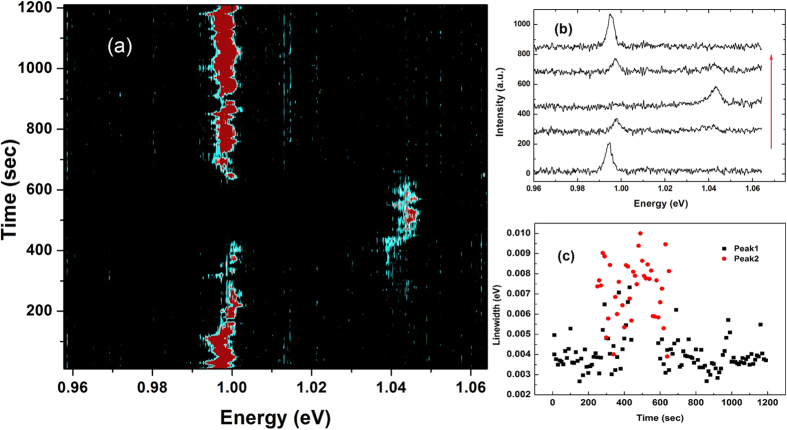
Temporal evolution of a single CNT. The data were collected by focusing laser beam on a single CNT and collecting the emission at 10 s intervals. Colors changing from black to red indicate increasing emission intensity. (**a**) 20 min temporal evolution as a function of photon energy. (**b**) Individual spectra taken from the temporal data shown in (**a**). (**c**) Plot of linewidth as a function of time evolution in (**a**).

Time traces of the emission spectra from individual CNTs were taken by confocally exciting their centres as shown in the temporal evolution of Fig. 4(a), where spectra were acquired every 10 seconds for 20 minutes. Example spectra are also shown in Fig. 4(b). The time separation between the spectra shown is approximately 100 to 300 seconds which demonstrates that the main peak from an (8, 6) nanotube at 0.993 eV can disappear and relocate to 1.043 eV, and then shift back to its original energy. There are reports which describe similar spectral diffusion behaviour arising from the QCSE^33^. This QCSE is not contradictory to our quantum dot-like excitonic observation owing to the non-Ohmic 0D phonon localization induced by aggregated PFO nanocrystals. Backscattering between the polymer nanocrystals on the tube would adjust its dielectric environment and thus break the tube into a series of quantum dot-like confined electronic states. Even though localized states form at cryogenic temperature, many electrons in the nanotube are still likely to be delocalized and diffuse along the tube axis. Figure 4(c) details the temporal evolution of the linewidth for the two peaks seen in Fig. 4(a). Peak 1 is at approximately 0.993 eV and peak 2 1.043 eV. Note the increase in linewidth seen in the region where spectral diffusion has caused the peak to change energy. Quantitatively, according to theoretical calculation^34^, to generate a 50 meV spectral diffusion, a surface electric field of more than 3 × 10^5^ V/cm is necessary, which can be produced by an electron at a distance over 15 nm. The nanocrystal is on the order of right size to form an electronic trap at the close vicinity of the localized excitonic state to create tens of meV spectral shift. Note the relatively long lifetime of the QD-like excitonic state at cryogenic temperature shifts the emission back to 0.993 eV.

## Conclusion

In conclusion, we report time-integrated photoluminescence measurements on *π*-conjugated polymer surfactant carbon nanotubes. We observe ultra-narrow linewidths down to 160 *μ*eV, which demonstrates an intrinsic excitonic behaviour. The corresponding longer dephasing time has been attributed to the CNTs detaching from environment introduced by polymer wrapping. We observe state filling effects in individual carbon nanotubes at 4.2 K which is consistent with a scenario of three dimensional confinement in quantum dots where the local energy landscape breaks the tube into zero-dimensional segments along a chain. The quantum dots in the chain emit up to four lines as the excitation power is increased. An exciton-phonon coupling model was used to fit the emission line. Multiple lines cannot be fitted by our model, but this again emphasizes the 3D confinement. Finally we discussed spectral diffusion resulting from exchange effects for the mobile carriers. Our experiments demonstrate that quantum dot-like states do exist in CNTs dependent upon the environment. We propose that much more detailed studies on exciton transport schemes are needed in order to promote the applications of carbon nanotubes in optoelectronic and quantum information devices.

## Methods

### Sample preparation

Firstly, 5–10 mg of the various polymers were added to 10–20 ml of an organic solvent (toluene, or cholorobenzene). To completely disperse the polymer, this mixture was treated in a sonic bath for about 60 mins. Next, the bought powdered CNTs were added to the mixture at an appropriate amount, which is normally around 5–10 mg. The material was subsequently treated in a high power ultrasonic disintegrate at a frequency of 23 kHz with a 10 micron amplitude for 15 mins. The disintegration breaks the CNT bundles into raw CNTs and therefore allows an interaction between individual CNTs with the dissolved polymer chains in the dispersion. Specifically, the PFO polymer here has a high selectivity towards HiPCO CNT with (8, 6) chirality. All polymers and CNT raw materials were used without further purification or processing in air.

### Measurement

A continuous wave Ti:Sapphire laser was used to excite the samples. The position of laser spot on the sample was controlled using a 100× microscope objective with N.A. 0.5 mounted on a sub-micron precision piezo-electric stage. The sample was placed in a Janis ST-500 microscope cryostat and the PL emission was detected using an InGaAs diode array mounted on an imaging 0.3 m spectrograph, having a spectral resolution of 100 *μ*eV at the emission energy of interest. All our measurements were performed near 4.2 K.

## Additional Information

**How to cite this article**: Wang, X. *et al.* Quantum dot-like excitonic behavior in individual single walled-carbon nanotubes. *Sci. Rep.* **6**, 37167; doi: 10.1038/srep37167 (2016).

**Publisher’s note**: Springer Nature remains neutral with regard to jurisdictional claims in published maps and institutional affiliations.

## Supplementary Material

Supplementary Information
