# Supplementary material for: Quantum dot-like excitonic behavior in individual single walled-carbon nanotubes
Source: Sci Rep. 2016 Nov 16;6:37167. doi: 10.1038/srep37167 (PMC5111057; doi:10.1038/srep37167)
Supplement: Supplementary Information [file srep37167-s1.doc]

# Electronic Supplementary Information for

# Quantum dot-like excitonic behavior in individual single walled-carbon nanotubes

## Xu Wang,^∗a,b^ Jack A. Alexander-Webber,^b^ Wei Jia,^b^ Benjamin P. L. Reid,^b^ Samuel D. Stranks,^b^ Mark J. Holmes,^b^ Christopher C. S. Chan,^b^ Chaoyong Deng,^b^ Robin J. Nicholas,^b^ and Robert A. Taylor^∗b^

^a^ College of Big Data and Information Engineering, Guizhou University, Huaxi, Guiyang, 550025, P. R. China; E-mail: sci.xuwang@gzu.edu.cn

^b^ Department of Physics, University of Oxford, Clarendon Laboratory, Parks Road, Oxford, OX1 3PU, U.K.; E-mail: r.taylor1@physics.ox.ac.uk

Figure SP1 shows two obvious higher energy peaks and a lower energy shoulder besides the main peak at 1.03eV. In the low excitation power regime (0.25mW/μm^2^ and below), only the main peak rises. When the excitation power is increased, two higher energy peaks appear and as the power increases further a fourth peak emerges at lower energy on occasion. This is also consistent with our calculations arising from the coupling of the two degenerate valleys at the K and Kʹ points, which can produce a splitting for the free excitons.


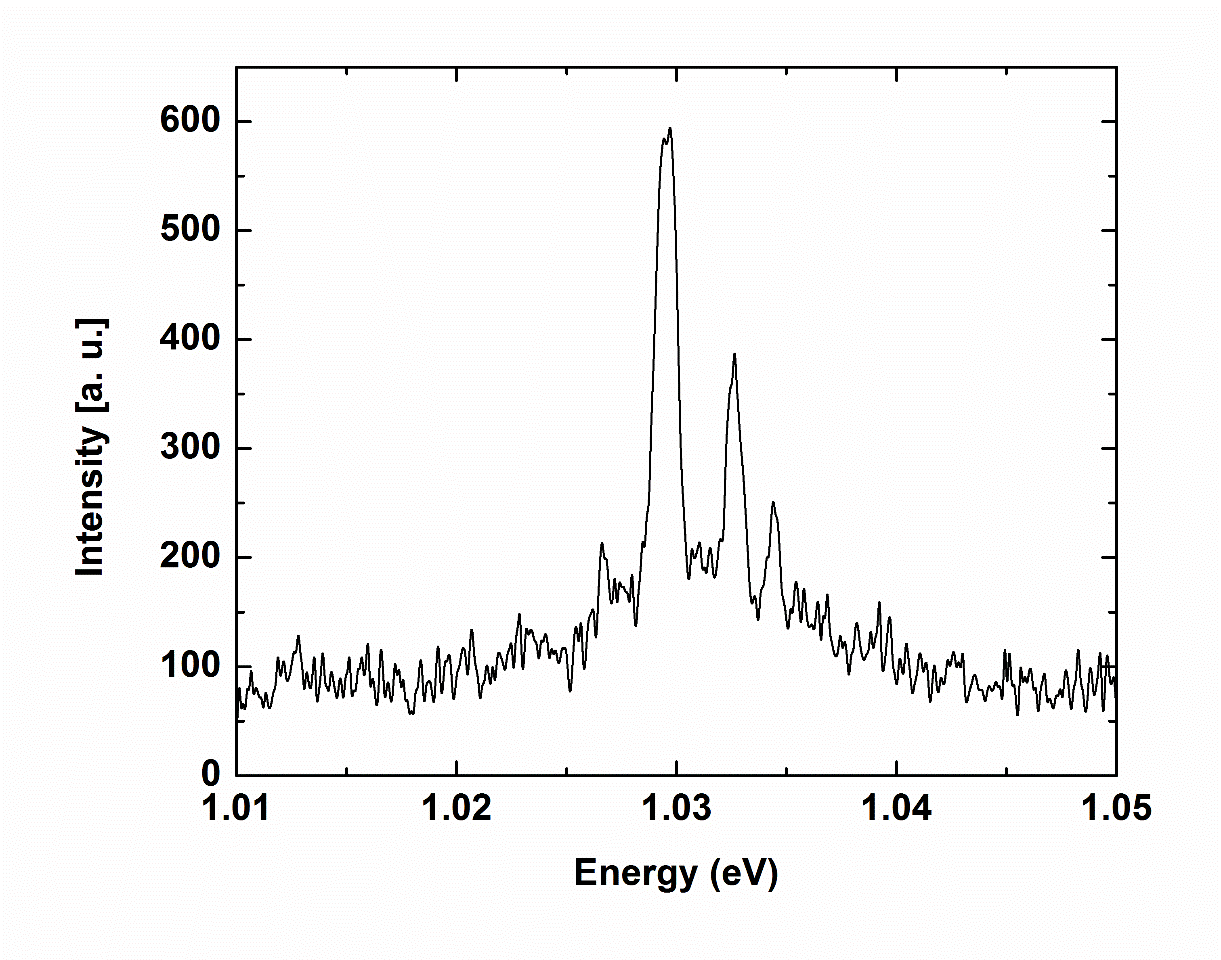


**Fig. SP1 A spectrum showing the state filling effect with one extra peak at lower energy side of the main peak at 1.03eV**

The PL lineshape is primarily governed by the interaction of excitons with 1D acoustic phonons, and can therefore be treated theoretically using an independent boson model. We assume a harmonic confinement localizes the exciton in CNTs with well-defined energy splitting ΔE≫k_B_T and this system is independent of the surrounding environment. Figure SP2 shows our fitting to experimental data. The redline fits well to PL from the PFO-HiPCO CNT. We use an exciton confinement length σ of 3nm and a tube length of 1500nm with an exciton effective mass of ~0.2. Thus our model works well with strongly confined nanotubes.


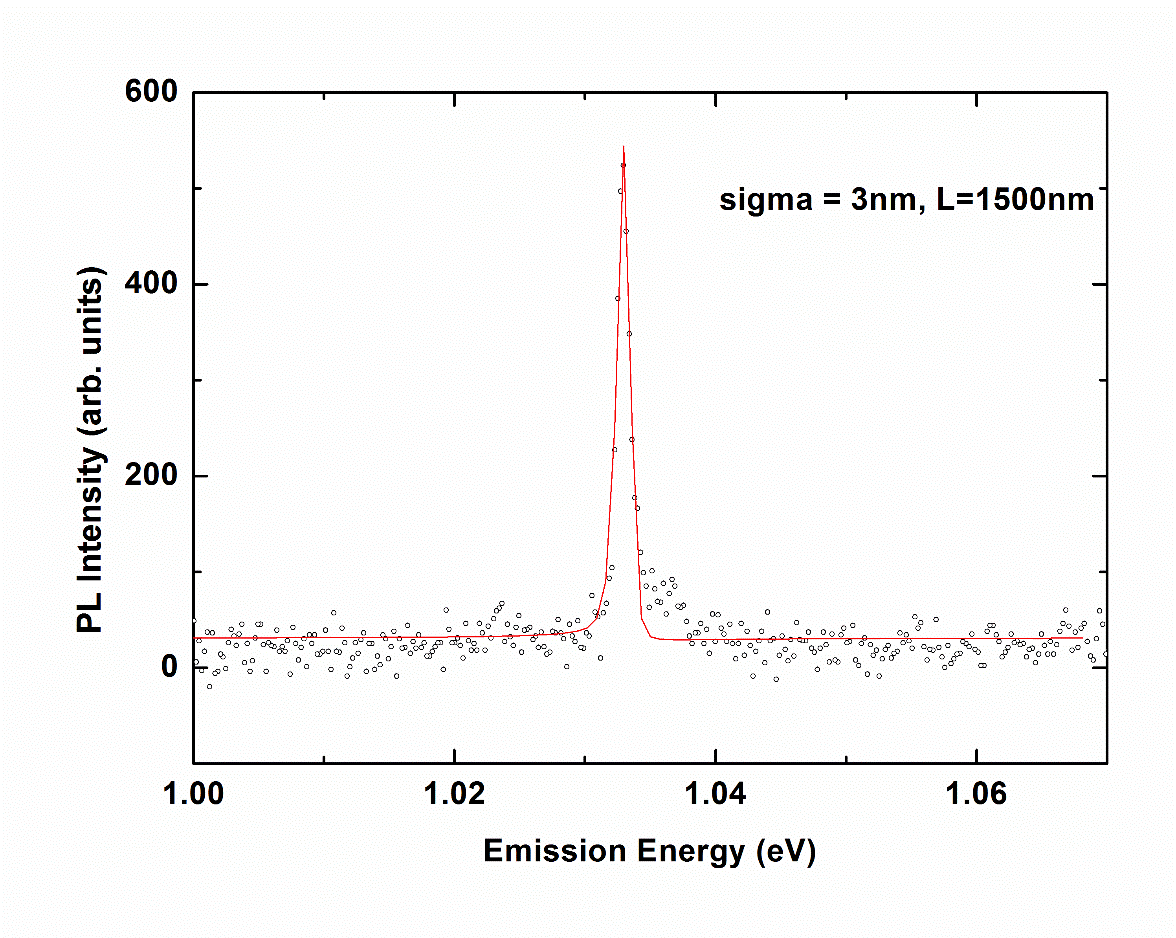
**Fig. SP2 Theoretical fit to a single peak of a CNT emission.**


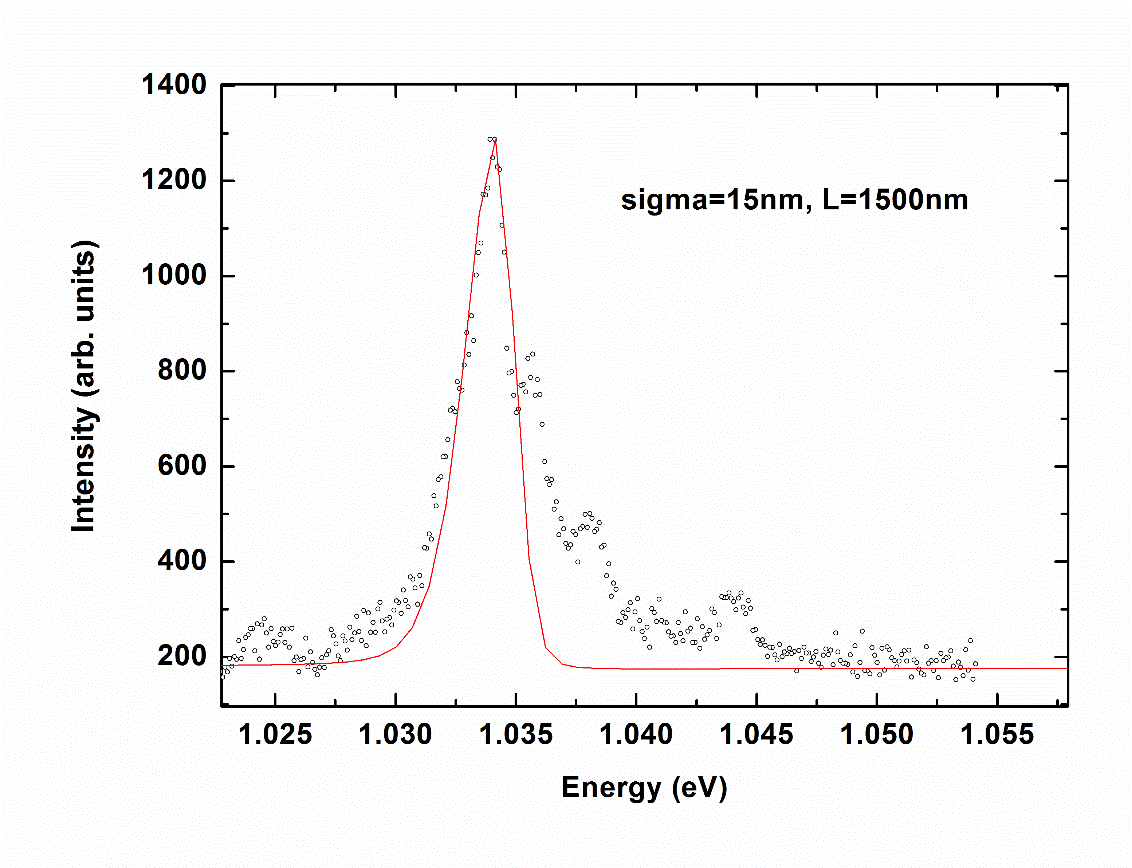


**Fig. SP3 Theoretical fit to multiple peaks as the excitation power is increased.**

However, Figure SP3 shows a fit of the theory to a multipeak spectrum. We have to adjust σ to 15nm, thus ΔE is no longer much larger than k_B_T, i.e. weak confinement. The experimental data cannot be fitted properly because of the higher energy peaks. Nonetheless, the Ohmic model provides evidence to support the assertion that the luminescence originates from quantum dot confinement. Where the equidistant peaks observed in Figure SP1 arise from different states of the same QD, and state filling effects will quench the confinement. This further supports the state filling model.

Figure SP4 shows two further (8,6) CNTs. As excitation power increases, the emergence of higher energy states are presented. Further reinforcing the state filling hypothesis set out in the main text.


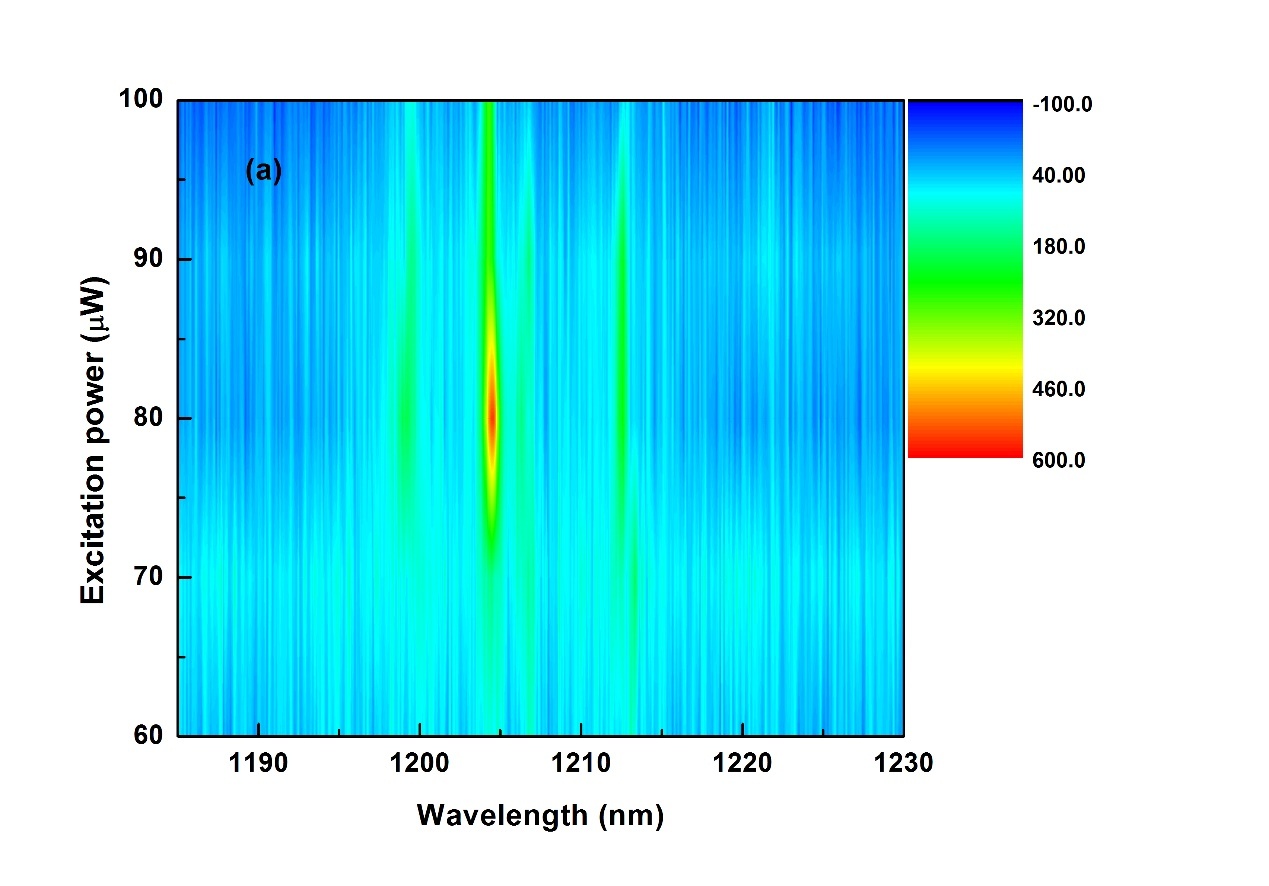


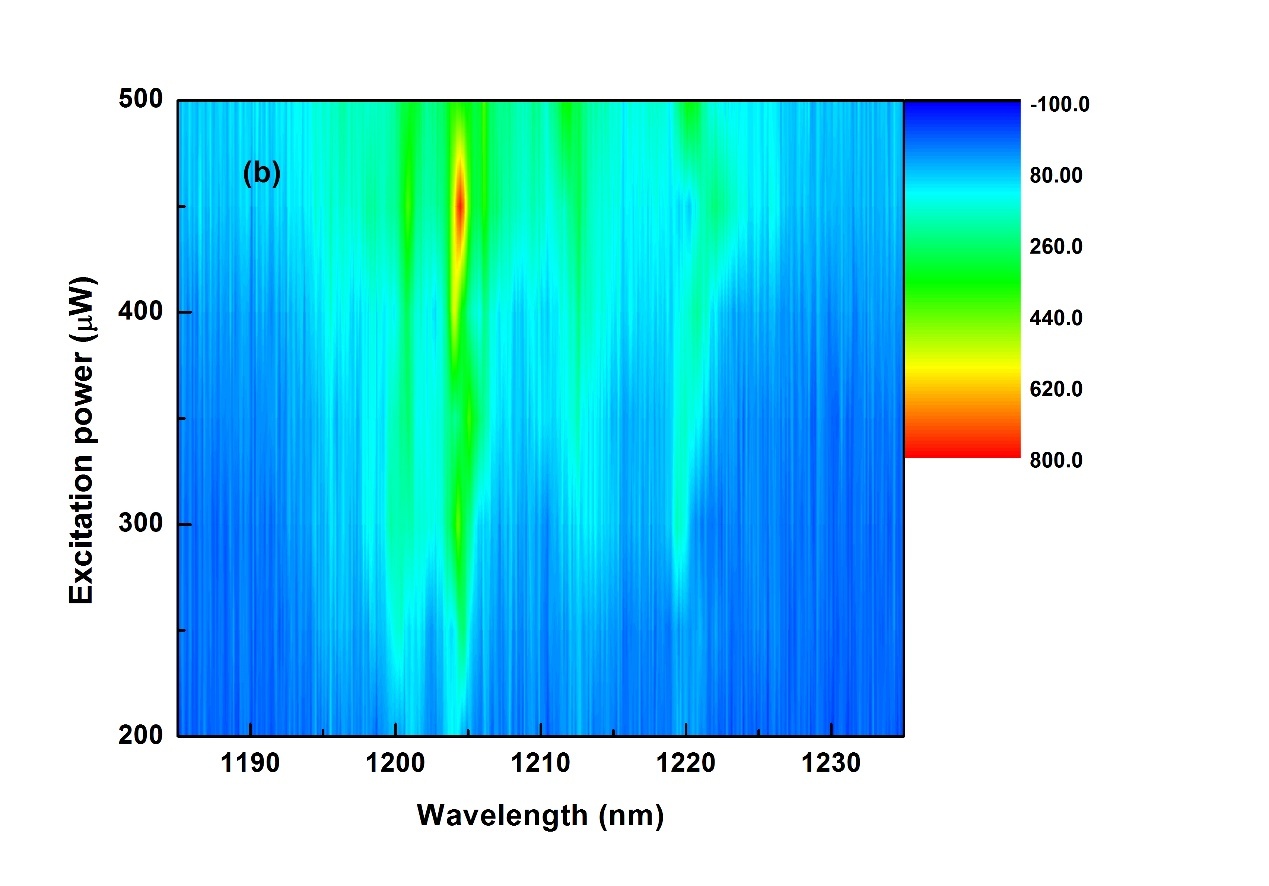


**Fig. SP4 Power dependence measurements for more individual (8,6) CNTs. (a) and (b) are plots of excitation power against emission wavelength. New states emerge with increasing excitation energy. Colors changing from blue to green to red indicate an increase in peak intensity.**


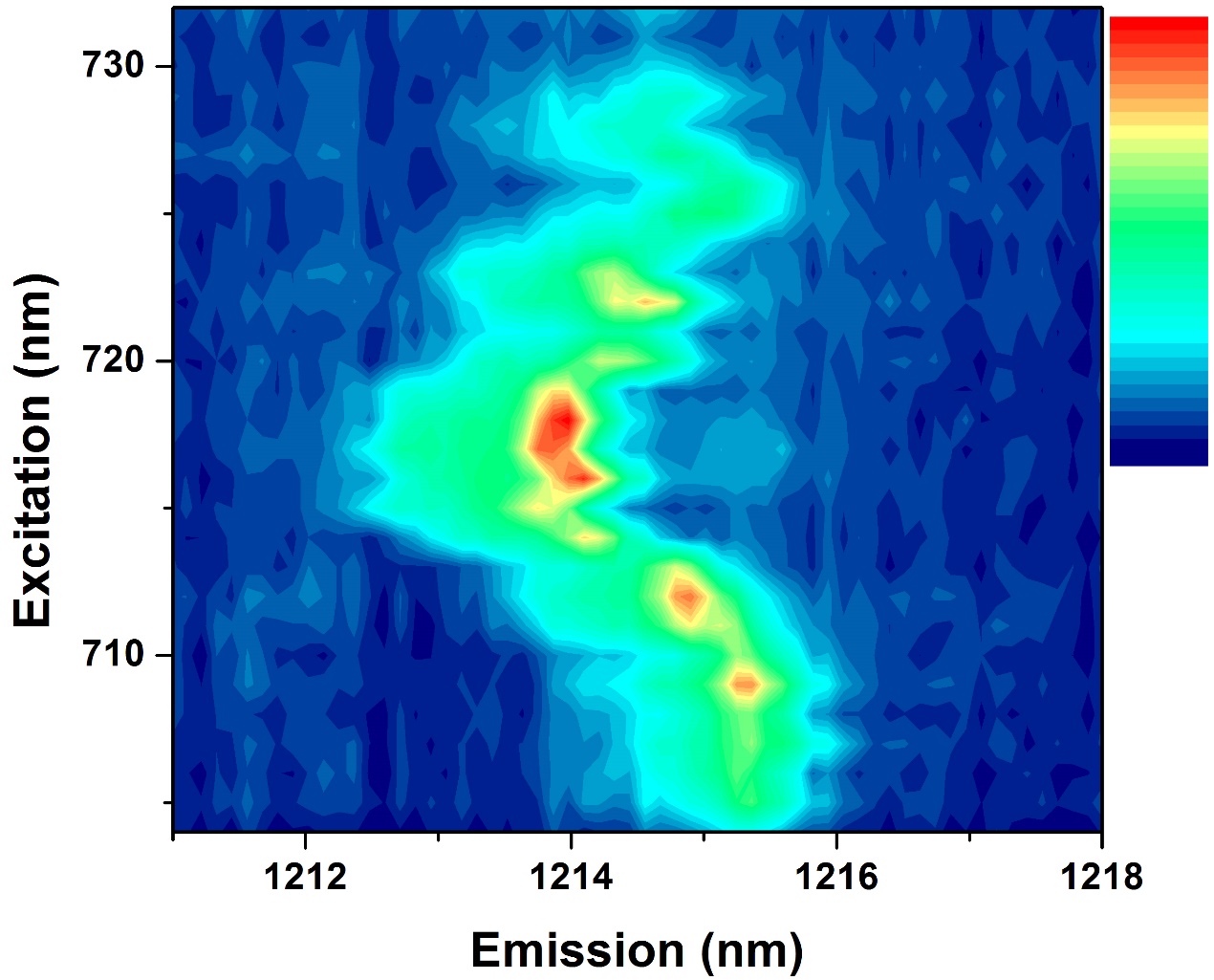


**Fig. SP5 PLE spectrum of a polymer CNT.**

We took many photoluminescence excitation (PLE) maps of the nanotube samples by varing excitation energy while collecting emission spectra. As shown in Figure SP5, color changing from blue to red representing increase of emission intensity. The linewidth of the E22 absorption (Excitation) remains broad about 50meV, due to ultrafast (130fs) depopulation of the E22 van Hove singularities (reference 20). Whereas the FWHM of E11 (Emission) is typically 2 orders of magnitude narrower. The narrowing is a particular property of emission from individual CNTs despite the fact that the ensemble of CNTs with the same chirality shows strong inhomogeneous broadening.

Finally, Figure SP6 shows the emission spectra recorded as excitation power increases. One can see clearly that the emission intensity saturates after 300μW, which is in accordance with Figure 3(c). Furthermore, as denoted by the three verticle lines, the distance between each peak is 2meV. The equal energy separation is also a characteristic of a state filling effect such as seen in compound semiconductor QDs.


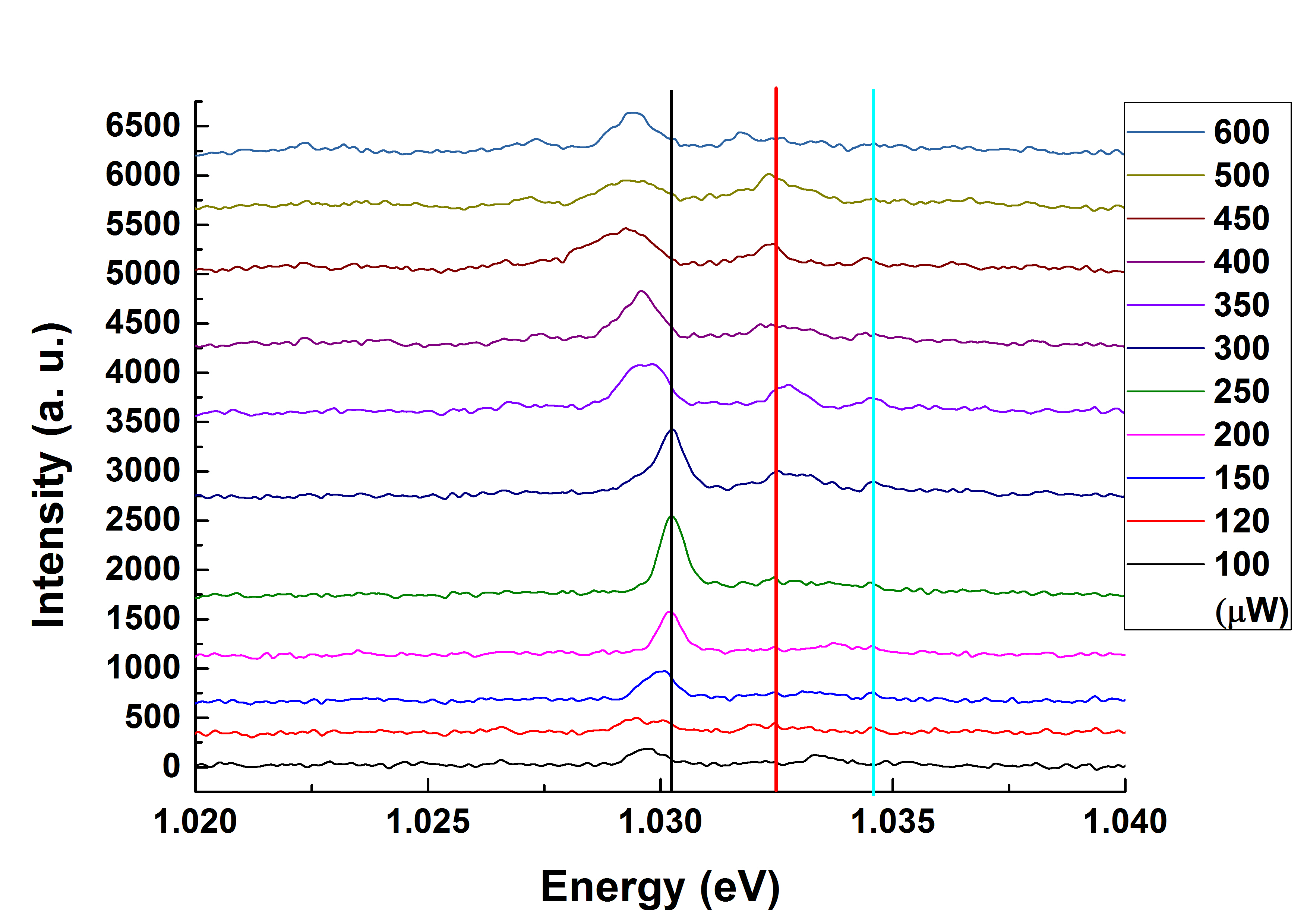


**Fig. SP6 Power dependence measurements for an individual (8,6) CNT. Three vertical lines denote the equal distance between each peak.**
